# Increased cholinergic contractions of jejunal smooth muscle caused by a high cholesterol diet are prevented by the 5-HT_4 _agonist – tegaserod

**DOI:** 10.1186/1471-230X-6-8

**Published:** 2006-02-23

**Authors:** Ronald Mathison, Eldon Shaffer

**Affiliations:** 1 Department of Physiology and Biophysics, University of Calgary, Calgary, AB, T2N 4N1, Canada; 2 Department of Medicine, Faculty of Medicine, University of Calgary, Calgary, AB, T2N 4N1, Canada

## Abstract

**Background:**

Excess cholesterol in bile and in blood is a major risk factor for the respective development of gallbladder disease and atherosclerosis. This lipid in excess negatively impacts the functioning of other smooth muscles, including the intestine. Serotonin is an important mediator of the contractile responses of the small intestine. Drugs targeting the serotonin receptor are used as prokinetic agents to manage intestinal motor disorders, in particular irritable bowel syndrome. Thus, tegaserod, acting on 5-HT_4 _receptor, ideally should obviate detrimental effects of excessive cholesterol on gastrointestinal smooth muscle. In this study we examined the effect of tegaserod on cholesterol-induced changes in the contractile responses of intestinal smooth muscle.

**Methods:**

The effects of a high cholesterol (1%) diet on the *in vitro *contractile responses of jejunal longitudinal smooth muscle from Richardson ground squirrels to the cholinergic agonist carbachol were examined in the presence or absence of tetrodrodotoxin (TTX). Two groups of animals, fed either low (0.03%) or high cholesterol rat chow diet, were further divided into two subgroups and treated for 28 days with either vehicle or tegaserod.

**Results:**

The high cholesterol diet increased, by nearly 2-fold, contractions of the jejunal longitudinal smooth muscle elicited by carbachol. These cholinergic contractions were mediated by muscarinic receptors since they were blocked by scopolamine, a muscarinic receptor antagonist, but not by the nicotinic receptor antagonist, hexamethonium. Tegaserod treatment, which did not affect cholinergic contractions of tissues from low cholesterol fed animals, abrogated the increase caused by the high cholesterol diet. With low cholesterol diet TTX enhanced carbachol-evoked contractions, whereas this action potential blocker did not affect the augmented cholinergic contractions seen with tissues from animals on the high cholesterol diet. Tegaserod-treatment removed the effects of a high cholesterol diet on neuronal muscarinic receptors, as the potentiating effect of TTX on carbachol-elicited contractions was maintained in these animals.

**Conclusion:**

A high cholesterol diet causes significant changes to cholinergic neurotransmission in the enteric nerves of the jejunum. The mechanisms by which these effects of cholesterol are reversed by tegaserod are unknown, but relate to removal of an inhibitory effect of cholesterol on enteric nerves.

## Background

In cholesterol gallstone formation, gallbladder smooth muscle develops reduced contractions, and the resulting impaired emptying leads to stasis. This dysfunction retains the cholesterol crystals that have nucleated from the supersaturated bile; the microcrystals agglomerate and stone growth follows [1,2]. Patients with gallstones also exhibit delayed small intestinal [3] and colonic [4] transit, which results in decreased enterohepatic cycling of bile salts [5] and so increases deoxycholic acid formation from cholic acid [6]. The deoxycholic acid and the reduced enterohepatic cycling adversely influence hepatic bile salt and cholesterol secretion and contribute to the formation of bile supersaturated with cholesterol.

A participatory role of the intestine in gallstone formation has also been established in animal models of gallbladder disease. The Richardson ground squirrel and the prairie dog on a high cholesterol diet develop lithogenic bile containing excess cholesterol relative to its solubilizing capacity [7,8]. Smooth muscle function of these animals is altered negatively: their gallbladders exhibit reduced contractile responses to CCK8 and acetylcholine [8], while small intestine transit and the cycle period of migrating myoelectric complexes are prolonged [9,10]. The basis for these intestinal motility changes is unknown, but might relate to the excess biliary cholesterol excreted into the duodenum and/or the hypercholesterolemic state of the animal model fed a high cholesterol diet.

From a therapeutic perspective, "prokinetic" agents, such as indoramin, an alpha-adrenergic antagonist, cisapride, a 5-HT_4_-agonist/5-HT_3_-antagonist, and erythromycin, a motilin agonist, promote small and large bowel transit, and enhance gallbladder emptying and so hinder gallstone formation [10,11]. In Richardson ground squirrels, cisapride reverses the defect in gallbladder contractility, enhances bile salt secretion and lowers cholesterol saturation [11]. Since cisapride is now known to cause cardiac rhythm disturbances [12], a new prokinetic agent, tegaserod, a partial 5-HT_4_-agonist [13], which enhances peristalsis, was approved for the treatment of patients exhibiting symptomatic irritable bowel syndrome (IBS) with constipation. IBS generally is associated with an increased risk of abdominal surgery, including cholecystectomy [14]. Hence, drugs used to treat this condition should not adversely affect gallbladder function. In a recent human study, tegaserod did not alter gallbladder contractility or the diameter of the common bile and hepatic ducts [15]. Nonetheless, the effects of tegaserod on small bowel function when there is a predisposition to gallstone formation are not known.

Since serotonin agonists contribute to the contractile responses of the small intestine [16,17] and are used to manage intestinal motor disorders [18,19], we postulated that tegaserod would modify cholesterol-induced changes in the contractile responses of intestinal smooth muscle. To test this hypothesis we examined the effects of tegaserod on cholinergic-induced contractions of the longitudinal smooth muscle of jejunal and ileal tissues obtained from Richardson ground squirrels that were maintained for 28 days on either a low (0.03%) or a high (1%) cholesterol diet.

## Methods

### Experimental animals

The University of Calgary Animal Care Committee approved the research protocol, which conforms to the guidelines of the Canadian Council on Animal Care. 76 Richardson ground squirrels (*Spermophilus richardsoni*) were trapped wild near Calgary, Alberta and were given ivermectin (Merial Canada Inc., Baie d'Urfé, QC, Canada) shortly after capture to eliminate nematode parasites. The animals were acclimatized for a minimum period of four weeks by caging them individually in thermoregulated rooms on a 12 h/12 h day/night light cycle with free access to a standard rat chow diet. Most animals adjusted well to captivity and gained weight, but 4 (7%) of them did not thrive or gain weight and were excluded from the study. After acclimatization, the animals were fed, under the same holding conditions for an additional four weeks, either a low cholesterol (normal) diet (0.03%, Dyets Inc., Bethlehem, PA, USA) or a high cholesterol diet (Dyets Inc.), comprising an identical chow enriched with 1% cholesterol by weight. During this diet period the animals were injected subcutaneously, twice daily, with either vehicle or 0.1 mg/kg of tegaserod (kindly supplied by Novartis Pharma, Basel, Switzerland). Tegaserod was dissolved in vehicle (100% 1-methyl-2-pyrrolidinone) immediately before use, and then diluted into 154 mM NaCl such that the final concentration of 1-methyl-2-pyrrolidinone was 2.7%. The vehicle-treated animals received 2.7% 1-methyl-2-pyrrolidinone (Sigma-Aldrich, St. Louis, MO, USA) in 154 mM NaCl. The animals were randomly divided into four groups, with 18 in each group and received the following combination of diet and drug: (1) vehicle + low cholesterol diet, (2) vehicle + high cholesterol diet, (3) tegaserod + low cholesterol diet, and (4) tegaserod + high cholesterol diet. The treatments were organized so that 2 animals from any one treatment group were used for experiments 28 days later. Gallbladder and hepatic bile cholesterol concentration and intestinal contractility were measured with all animals.

### Cholesterol concentrations in hepatic and gallbladder bile

For collection of common duct bile animals were fasted for 14 hours, and then anesthetized and maintained on a low concentration (1.5 to 2%) isoflurane (Ayerst Laboratories, Montreal, QC, Canada). At laparotomy the cystic duct was exposed and ligated, the gallbladder removed and the bile collected for subsequent analysis. The common bile duct was cannulated with a polyethylene catheter (PE50), and hepatic bile was collected for two one-hour periods. During this period 154 mM NaC1 was infused continuously IV at 2.5 mL/h via a femoral venous cannula for fluid replacement.

Cholesterol was measured using the Liebermann-Burchard reaction (Cholesterol/Cholesteryl Ester Quantitation Kit™ of Biovision; Mountain View, CA, USA).

### Intestinal contractility *in vitro*

At the end of the collection period for hepatic bile the intestine was removed to evaluate contractile responses of smooth muscle [9]. Four 2 cm lengths of intestine were obtained in each animal, two from the proximal jejunum at a point 5 cm distal to the ligament of Treitz, and two from the distal ileum at a location 15 cm proximal to the ileocecal valve. Without occluding the lumen, 5-0 silk threads were tied to opposite ends of each segment with one end fixed to the bottom of a 20 mL organ bath, and the other to an isometric force-displacement transducer (FT03; Grass Telefactor, West Warwick, RI, USA)). This montage measured the contractile response of the longitudinal smooth muscle layer. The baths, maintained at a temperature of 35°C, were filled with a modified Krebs solution of the following composition (in mM): NaC1 103, KC1 4.7, MgC1_2 _1.13, NaHCO_3 _25, NaH_2_PO_4 _1.15, CaC1_2 _2.56, D-glucose 2.8, sodium pyruvate 4.9, sodium fumarate 2.7, and sodium glutamate 4.9: at pH 7.4, continuously gassed with a mixture of 95% O_2 _and 5% CO_2_.

The tissues were stretched to 1 gm of tension, and equilibrated for 40 min with a change of physiological buffer every 10 min, and appropriate adjustment of tension. After equilibration, the longitudinal intestinal segments underwent cumulative dose-responses (10^-8^M to 10^-4^M) to carbamylcholine chloride (carbachol; Sigma-Aldrich), performed either in the presence or absence of either 10^-7^M tetrodotoxin (TTX; Sigma-Aldrich), or 10^-5^M hexamethonium (Sigma-Aldrich). The TTX permitted evaluation of smooth muscle contractility in the absence of neural influences, and hexamethonium, a selective nicotinic antagonist, was used to determine the contribution of nicotinic receptors to the increased tone. With each animal a jejunal or ileal segment was preincubated with either TTX or hexamethonium for 10 min, whereas the other untreated segments served as carbachol controls. Scopolamine was added to the tissues after completion of the cumulative dose-response for carbachol in all animals groups. At the end of each experiment, the tissue preparations were lightly blotted and weighed. The contractile response for each preparation was calculated as stress (Newtons/m^2^), normalized to the cross-sectional area of the longitudinal smooth muscle layer as previously described [9].

### Data analysis

The results are presented as the mean ± SEM. The statistical functions used that associated with Excel (Microsoft Office XP, Redmond, WA, USA). Comparisons between the different concentrations of carbachol for two groups were made using the unpaired Student's t-test. With cholesterol measurements one-way analysis of variance was applied, with the differences between groups identified using the unpaired Student's t-test. Statistical values reaching probabilities of p < 0.05 were considered significant.

## Results

### Bile cholesterol

For vehicle-treated animals on a low cholesterol diet the concentration of cholesterol in gallbladder and hepatic duct bile were 2.84 ± 0.37 (n = 14) and 0.85 ± 0.14 (n = 12) mM, respectively. Tegaserod treatment of low cholesterol diet animals did not modify cholesterol concentrations in the gallbladder (2.83 ± 0.38 mM; n = 14) or the hepatic duct (0.67 ± 0.11 mM; n = 13) relative to vehicle-treated animals. Feeding the animals a high cholesterol diet for 28 days significantly increased the bile cholesterol concentrations to 4.33 ± 0.47 (n = 15) and 1.91 ± 0.30 (n = 14) mM in the gallbladder and hepatic duct, respectively. Treating animals on the high cholesterol diet with tegaserod did not modify cholesterol concentration in gallbladder bile (4.04 ± 0.58 mM; n = 14), but reduced it in hepatic duct bile (1.29 ± 0.19 mM; n = 13) to concentrations significantly lower than that seen in the vehicle-treated, high cholesterol animals. These concentrations of hepatic duct bile were higher than those seen with tegaserod-treated, low cholesterol animals.

### Phasic and tonic contractions of intestinal segments

Relative to control animals neither the high cholesterol diet nor the tegaserod treatment, alone or in combination, modified basal and carbachol-induced changes in the phasic contractions of the jejunal and ileal segments. In addition, neither TTX nor hexamethonium altered the phasic contractile activity of the intestinal tissues in any of the animal groups. However, as discussed in the following sections tonic contractile responses to cholinergic stimulation with carbachol where significantly altered by the cholesterol diet and tegaserod treatment, and these alterations were further modified by TTX and hexamethonium.

### Cholinergic contractions of jejunal longitudinal smooth muscle

**Figure 1:**
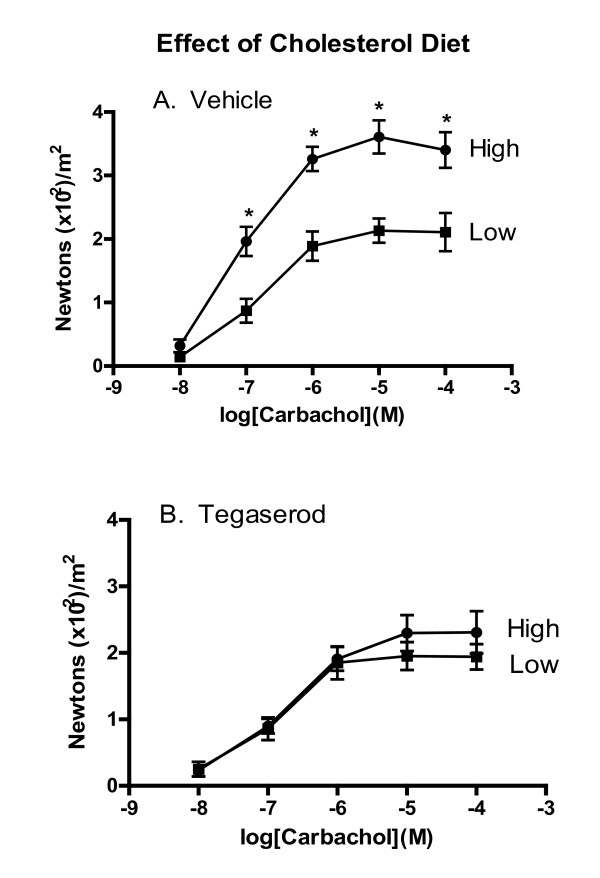
**Cholesterol diet and cholinergic contractions of the longitudinal muscle of the jejunum**. Effects of cholesterol diet on cholinergic (carbachol) – elicited contractions of the jejunum for ground squirrels on a trace (0.027%; Low) vs. enriched (1%; High) cholesterol diet for animals treated with either vehicle (A) or tegaserod (B). Cumulative dose-response curve to carbachol were constructed. * High greater than Low (P < 0.05); N = 8–9.

Isolated jejunal segments from the ground squirrels on a low cholesterol diet responded in a dose-dependent manner to carbachol with increasing tonic contractions to a maximal force of 2.1 ± 0. × 10^2 ^Newtons/m^2 ^at 10^-4^M carbachol (Figure 1A). In contrast, animals on the high cholesterol diet receiving the vehicle exhibited significantly greater responses to concentrations of carbachol of 10^-7^M and higher, and showed a maximal response of 3.6 ± 0.3 (× 10^2^) Newtons /m^2^.

After 4 weeks of tegaserod treatment of animals on a low cholesterol diet, 10^-4^M carbachol generated a maximal force of 1.9 ± 0.2 × 10^2 ^Newtons/m^2 ^(Figure 1B), which was similar to the response in vehicle-treated animals. In contrast, jejunal tissues from tegaserod-treated animals on the high cholesterol diet did not exhibit any heightened contractions to carbachol; the maximal response of 2.3 ± 0.3 × 10^2 ^Newtons/m^2 ^was 60% smaller than that seen with vehicle-treated animals on the high cholesterol diet.

### Effect of action potential blockade on carbachol-evoked contractions

**Figure 2:**
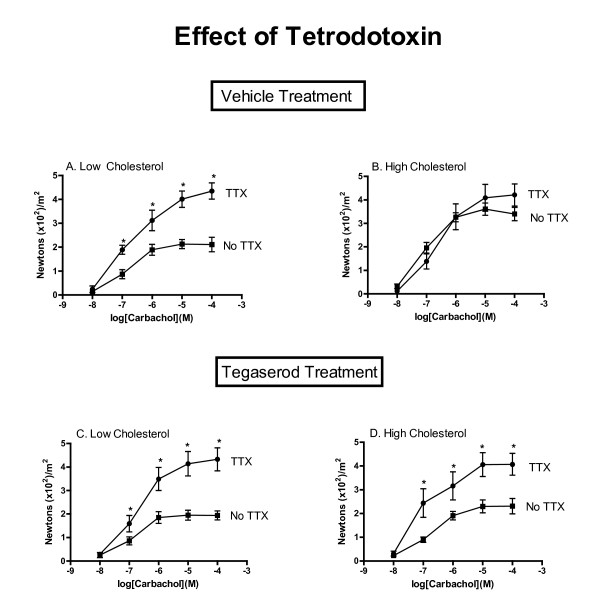
**Action potential blockade and contractions of the longitudinal muscle of the jejunum**. Effects of tetrodotoxin (TTX) on cholinergic contractions of the jejunum of ground squirrel fed either a low (0.027%; A & C) or a high (1%; B & D) cholesterol diet. The animals were treated with either vehicle (A & B) or tegaserod (C & D). The action potential blocker tetrodotoxin (TTX; 10^-7^M) was added to the jejunal tissue segments 10 min before constructing a cumulative dose-response curve to the cholinergic agonist, carbachol. * TTX greater than No TTX (P < 0.05). N = 8–9.

**Figure 3:**
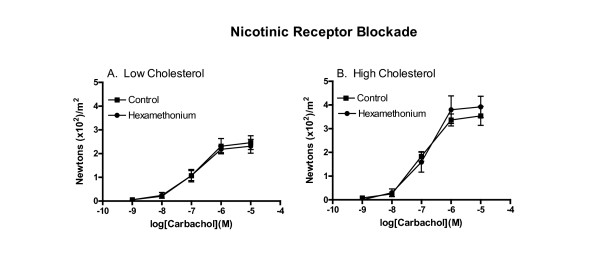
**Antagonism of nicotinic receptors and contractions of the longitudinal muscle of the jejunum**. Absence of an effect of the nicotinic receptor antagonist, hexamethonium (10^-5^M) on cholinergic contractions of the jejunum of vehicle-treated ground squirrel fed either a low (0.027%; A) or a high (1%; B) cholesterol diet. Hexamethonium was added to the jejunal tissue segments 10 min before constructing a cumulative dose-response curve to the cholinergic agonist, carbachol. N = 5–6.

To determine if the cholinergic contractions of the jejunal longitudinal smooth muscle had a neural component, contractions of jejunal smooth muscle were measured after the addition of TTX, a blocker of action potential conduction. In the presence of TTX the dose-response relationships to carbachol on tissues from the vehicle-treated animals on a low cholesterol diet increased approximately 2-fold to 4.4 ± 0.4 (× 10^2^) Newtons/m^2 ^(Figure 2A). Thus, the carbachol-produced contractions of jejunal smooth muscle reflects the net action of two processes; a direct contractile action on smooth muscle, and a relaxation/inhibition caused by activation of intramural nerves, a process abrogated by TTX. Since the nicotinic receptor antagonist, hexamethonium, did not increase carbachol-elicited contractions (Figure 3A), the potentiation of the cholinergic contractions after TTX exposure was independent of neuronal nicotinic receptors. Scopolamine, a muscarininc antagonist, used at a concentration of 10^-5^M, totally blocked the contractile actions of carbachol (data not shown), and also abolished spontaneous contractions.

Animals on the low cholesterol diet treated with tegaserod, like the vehicle-treated controls, exhibited increased contractile responses to carbachol in the presence of TTX (Figure 2C). Hexamethonium did not modify carbachol-elicited contractions (Figure 3B). As before, scopolamine (10^-5^M) totally blocked any contractile response to carbachol (not shown). Thus, with tegaserod treatment carbachol contractions of jejunal smooth muscle from low cholesterol diet animals also reflects the net action of two processes; a direct contractile action on smooth muscle and a relaxant action caused by activation of intramural nerves.

**Figure 4:**
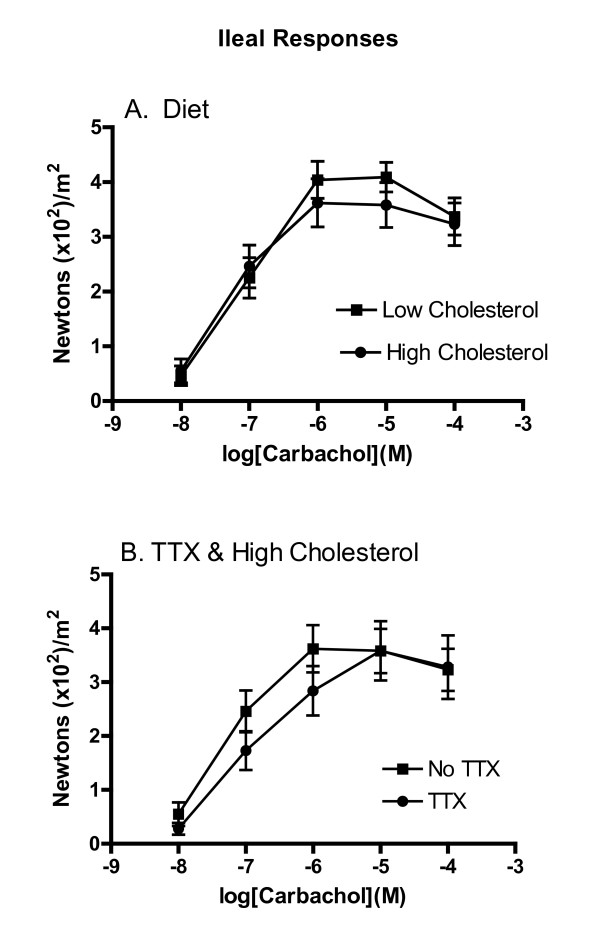
**Cholinergic contractions of ileal longitudinal smooth muscle in response to a high cholesterol diet and action potential blockade**. (A) Effects of cholesterol diet on cholinergic (carbachol) – elicited contractions of the ileum for ground squirrels on a trace (0.027%; Low) vs. enriched (1%; High) cholesterol diet for animals treated with vehicle. (B) Effects of tetrodotoxin (TTX) on cholinergic contractions of the ileum of ground squirrels fed a high (1%) cholesterol diet and treated with vehicle. The action potential blocker tetrodotoxin (TTX; 10^-7^M) was added to the jejunal tissue segments 10 min before constructing a cumulative dose-response curve to the cholinergic agonist, carbachol. N = 8–9.

Ileal contractions elicited by carbachol were not modified by the high cholesterol diet (Figure 4A), TTX (Figure 4B) or hexamethonium (not shown), indicating that nerve-dependent potentiation of smooth muscle contractions to carbachol occurred exclusively in the jejunum. Consequently, only the contractile responses to the jejunum are presented.

### Interactions between cholinergic contractions, action potential blockade and a high cholesterol diet

In contrast to the animals on the low cholesterol diet, cholinergic contractions of tissues from animals on the high cholesterol diet were not potentiated after exposure to TTX (Figure 2B). Since carbachol contractions, in the presence of TTX, were of a similar magnitude in tissues obtained from both low and high cholesterol diet animals, the high cholesterol diet probably increases carbachol-induced contractions by removing a cholinergic neurally-mediated inhibition of tonic smooth muscle contractions. This inhibition is muscarinic receptor-mediated since hexamethonium did not alter carbachol-evoked contractions, even in cholesterol-fed animals (Figure 3B).

For animals on a high cholesterol diet and treated with tegaserod a significant potentiation of the carbachol-elicited contractions was seen following exposure to TTX (Figure 2D). This potentiation is comparable to that seen with tissues from low cholesterol diet animals receiving either vehicle or tegaserod. Thus, tegaserod treatment restores a component of carbachol that was abrogated by the high cholesterol diet.

## Discussion

This study reveals that a high cholesterol diet, fed for 28 days, modifies the properties of cholinergic contractions of jejunal smooth muscle through modulation of cholinergic receptors located on enteric nerves. This action of the high cholesterol diet was prevented by treating the animals with tegaserod.

A high cholesterol diet has diverse effects on cholinergic-mediated smooth muscle contractions of different tissues. With few exceptions [20] a high cholesterol diet is reported to decrease, consequent to gallstone formation [21], the contractile responses of gallbladder smooth muscle to acetylcholine receptor activation in human [21], dog [22] and ground squirrels [8,9]. Few studies have examined the effects of a high cholesterol diet on intestinal smooth muscle contractions, but for the ileum either no effect [[8]; this study Figure 4], or increased contractions [9] have been reported. With the jejunum a high cholesterol diet increases smooth muscle contractile responses to cholinergic agonists [9], and the present study suggests that dietary cholesterol inhibits neuronal muscarinic receptors in the jejunum, allowing for expression of enhanced direct muscarininc receptor stimulated-contractions of smooth muscle. The reasons for differential responses of jejunal and ileal smooth muscle to cholinergic stimulation are not known, but differences in the local luminal concentrations of cholesterol metabolites known to modify cholinergic receptors may be an underlying cause. Taurocholate, which impairs cholinergic contractions of the gallbladder [23,24] is four times more concentrated in gallbladder bile than in hepatic bile [25]. The glycine and taurine conjugates of deoxycholic acid act as cholinergic muscarinic receptor antagonists [26], and their concentrations are possibly increased in the hepatic bile of cholesterol-fed animals. The absence of an effect of the high cholesterol diet on cholinergic responses in the ileum may reflect reduced concentrations of cholinergic modifying bile salts consequent to their enhanced absorption by the ileum relative to the jejunum [27].

Cholesterol is an essential membrane component serving as a cofactor for signalling molecules and as a precursor for steroid hormones. Cholesterol influences many of the biophysical properties of membranes, and depending on the receptor, cholesterol influences the affinity, binding capacity and signal transduction [28]. When inappropriately regulated or excessive, by altering plasma membrane structure-function, cholesterol can play a crucial role in many diseases [28]. Cholesterol's participation in cardiovascular disease is well established, and some neurological [29], immunological [30,31] and gastrointestinal disorders have cholesterol-related components. Cholesterol also has established roles in gallbladder disease [21,32] and modifies other gastrointestinal functions. Although acute exposure to cholesterol decreases pressure in rabbit intestinal loops [33] and reduces nitrergic relaxation of the rabbit sphincter of Oddi muscle [34], most cholesterol-related abnormal events probably result from prolonged exposure to this lipid, and its associated metabolites. In the current study animals were fed a high cholesterol diet for 28 days as with previous studies [9-11], but the time course for the modification of cholinergic receptor function by the cholesterol diet is not known, and acute effects cannot be excluded.

A nervous system-dependent component to cholinergic agonist contractions of the intestinal tissues from Richardson ground squirrels was revealed. Since TTX removed an inhibitory component of cholinergic contractions of the jejunum from animals on a low cholesterol diet (Figures 2A and 2C), a part of carbachol-elicited contractions, in low cholesterol dieted animals, are neuronally mediated. Given that carbachol is a mixed muscarinic-nicotinic receptor agonist [35], the neuronal component to cholinergic contractions could be mediated by either nicotinic or muscarininc receptors on neurons and nerve terminals [36-41]. Since hexamethonium, unlike TTX, did not modify carbachol contractions (Figure 1B) the potentiation of the carbachol-elicited contractions did not involve nicotinic receptors, but rather neuronal muscarinic receptors. This modulation by TTX of carbachol-elicited contractions was not evident in the ileum (Figure 4B), and thus either neuronal muscarinic receptors are absent, or possibly obscured, in this intestinal segment by the simultaneous activation of inhibitory and stimulatory receptors, or, as discussed above, the cholesterol metabolites causing the changes in the jejunum are not present in sufficient concentrations in the more distal segments of the small intestine.

In the small intestine 5-HT_4 _receptors, targets for tegaserod action are found exclusively on enteric nerves [43]. The 5-HT_4 _receptors, localized to cholinergic nerves, are involved in the contraction of smooth muscle from canine small and large intestines [43,44] and human large intestines [43]. Mucosal release of 5-HT with activation of 5-HT_4 _receptors on sensory neurons that relay via enteric interneurons to cholinergic motor neurons regulates the peristaltic reflex [45-48]. The rationale for the therapeutic use of agents acting at 5-HT_4 _receptors to modulate visceral hypersensitivity supposes that continuous mucosal stimuli cause auto-inhibition and desensitization of 5-HT_4 _receptors [49], and intramural sensory pathways [50]. We did not investigate this sensory-effecter pathway for smooth muscle activation, but rather we examined, using a regimen of drug administration used therapeutically, the actions of tegaserod on cholinergic receptor activation with carbachol. This 28 day treatment with tegaserod removed the potentiating effects of cholesterol diet on cholinergic contractions of the jejunum (Figure 1B) due to the masking of enteric nerve dependent-mediated inhibition of jejunal contractions (Figures 1B and 2D). These effects of tegaserod may also have contributions from unexplored variables such as changes in cholesterol metabolism and contraction-dependent reflex-induced release of 5-HT from enteric nerves.

## Conclusion

Maintaining ground squirrels on a high cholesterol diet for 4 weeks resulted in the removal of an enteric nervous system-dependent, muscarinic suppression of cholinergic contractions of jejunal smooth muscle. This cholesterol-mediated inhibition of muscarinic neuronal transmission was prevented by tegaserod, a 5-HT_4_-partial agonist.

## Abbreviations

5-HT – serotonin; CCK8 – cholecystokinin-octapeptide; CSI – cholesterol saturation index; IBS – irritable bowel syndrome; TTX – tetrodotoxin

## Competing interests

The author(s) declare that they have no competing interests.

## Authors' contributions

All authors participated in study design and read and approved the final manuscript. ES initiated the study and developed the protocol. RM coordinated the study, performed experiments, analyzed the data with statistical analysis and prepared the manuscript in conjunction with ES.

## Pre-publication history

The pre-publication history for this paper can be accessed here:
